# Methylmercury Causes Neurodegeneration and Downregulation of Myelin Basic Protein in the Spinal Cord of Offspring Rats after Maternal Exposure

**DOI:** 10.3390/ijms23073777

**Published:** 2022-03-29

**Authors:** Diane Cleydes Baía da Silva, Leonardo Oliveira Bittencourt, Daiane Claydes Baia-da-Silva, Victoria Santos Chemelo, Luciana Eiró-Quirino, Priscila Cunha Nascimento, Márcia Cristina Freitas Silva, Marco Aurelio M. Freire, Walace Gomes-Leal, Maria Elena Crespo-Lopez, Rafael Rodrigues Lima

**Affiliations:** 1 Laboratory of Functional and Structural Biology, Institute of Biological Sciences, Federal University of Pará, Belém 66075-110, PA, Brazil; dianecleydes@gmail.com (D.C.B.d.S.); leo.bittencourt25@gmail.com (L.O.B.); daiane.silva@ics.ufpa.br (D.C.B.-d.-S.); vicchemelo@gmail.com (V.S.C.); guimaraeseiro@gmail.com (L.E.-Q.); priscilacunha.n28@gmail.com (P.C.N.); marciaf@ufpa.br (M.C.F.S.); 2 Graduate Program in Health and Society, University of the State of Rio Grande do Norte, Mossoró 59610-210, RN, Brazil; freire.m@gmail.com; 3 Laboratory of Experimental Neuroprotection and Neuroregeneration, Unity of Morphophysiology, Federal University of Western Pará, Santarém 68040-470, PA, Brazil; wgomesleal@nervecell.org; 4 Laboratory of Molecular Pharmacology, Institute of Biological Sciences, Federal University of Pará, Belém 66075-110, PA, Brazil; maria.elena.crespo.lopez@gmail.com

**Keywords:** methylmercury, spinal cord, neurodegeneration, lactation, pregnancy

## Abstract

Methylmercury (MeHg) is one of the most dangerous toxic pollutants spread throughout the earth. Chronic MeHg intoxication by contaminated food ingestion is the most common threat to human health, including impairment to the developing fetus. The present study aims at investigating the effects of maternal exposure to MeHg during gestation and lactation on the spinal cord of offspring. Pregnant rats received oral doses of MeHg (40 μg/kg/day) over a period of 42 days (21 gestation and 21 lactation). Control animals received the vehicle only. Total mercury concentration was measured in blood samples from offspring collected at the 41st postnatal day. Counting of motor neurons and immunoreactivity for myelin basic protein (MBP) were assessed in the spinal cords in both control and MeHg-intoxicated animals. Our results showed that MeHg promoted an increase in blood Hg levels. In addition, it caused a reduction in the number of spinal cord motor neurons as well as decreased MBP immunoreactivity in the cervical, thoracic and lumbar segments. Our present findings suggest that MeHg intoxication during rat pregnancy and lactation is associated with a pattern of motor neuron degeneration and downregulation of myelin basic protein in different segments of a developing spinal cord. Further studies are needed to establish the effect of MeHg intoxication in both young and adult rats.

## 1. Introduction

Mercury is a toxic metal widely found in the environment due to natural factors, such as biomass burning, and anthropogenic factors, such as industrial activity and gold mining [1,2]. In addition, mercury is found in various chemical forms, classified as elemental (Hg^0^), inorganic species, such as mercury chloride (HgCl_2_), and organic species, such as methylmercury (MeHg) [1]. Since its above-cited employment in gold mining, mercury remains a serious environmental contaminant in the Amazon basin [3,4].

MeHg can be generated in the environment through the methylation of inorganic mercury by sulfur-reducing bacteria found in aquatic ecosystems, causing mercury methylation [2]. This organometallic compound undergoes biomagnification and bioaccumulation in several fish species, with significant levels of MeHg, which mainly affects riverine communities [2,5,6]. The mercury and their derivatives have special tropism by the central nervous system (CNS). According to Ruggieri et al. [7], the neurotoxicity of MeHg during prenatal care is harmful to the development of the CNS, due to the susceptibility of the brain during its maturation period, caused by enzymatic and metabolic disorders that affect cognitive and motor functions.

The spinal cord (SC) is part of the CNS and is located within the spinal canal, possessing nerves that leave the spine through descending tracts, which are of fundamental importance to carry commands to the peripheral parts of the body [8]. The motor functions are carried out by motor nerve fibers from the SC ventral roots, which control the voluntary muscles of the limbs and trunk [9]. Thus, considering motor functions, several preclinical lines of evidence associate the motor damage caused by MeHg intoxication with CNS structures, including the motor cortex [10].

There is evidence that exposure to MeHg causes neurological alterations in children at different stages of development [7,11]. Studies in animal models have demonstrated the motor changes in rats and mice [12,13]. Nevertheless, there are no studies investigating whether the effects of MeHg exposure during the intrauterine period and lactation persist over the SC maturation of offspring rats. In this study, we aimed to investigate the effects of MeHg intoxication during intrauterine and lactation periods on the motor neuron density and integrity of white matter tracts in the spinal cord of offspring rats.

## 2. Results

### 2.1. Offspring Details

During the experimental period when the females were pregnant, there were no abortions or losses of animals. The six pregnant rats gave birth to 36 rats—15 males and 21 females. To conduct the rest of the analyses, we considered only male rats to avoid possible size variations between males and females that could bias the morphometric analysis.

### 2.2. MeHg Exposure during Pregnancy and Lactation Increased Total Mercury Levels in the Offspring Blood

**Figure 1:**
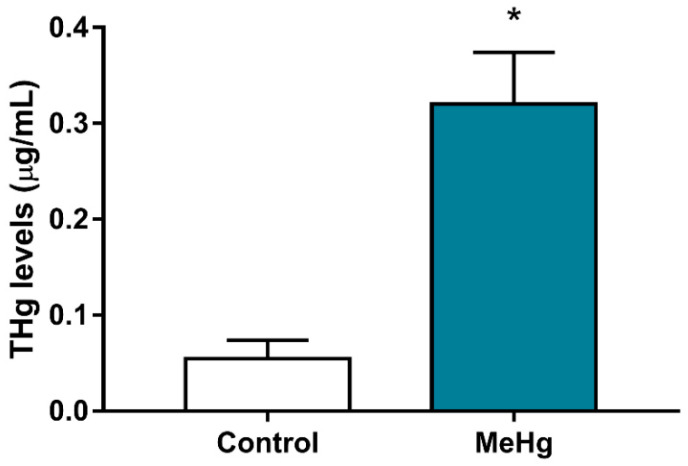
Exposure to MeHg during the gestation and lactation periods increases THg in the offspring (samples collected 21 days after 42 days of dosing). Results are expressed as the mean ± standard error of the mean (μg/mL). (*n* = 7–8 animals per group). * *p* < 0.05, Student’s *t*-test.

The total mercury (THg) levels present in the blood of offspring exposed to MeHg during the gestation and lactation periods were significantly higher (0.3205 ± 0.007638 μg/mL, when compared with the control group (0.055 ± 0.007638 μg/mL) (*p* < 0.0367) (Figure 1).

### 2.3. MeHg Exposure during Intrauterine and Perinatal Period Induces Neuron Degeneration in the Spinal Cord of Offspring Rats

**Figure 2:**
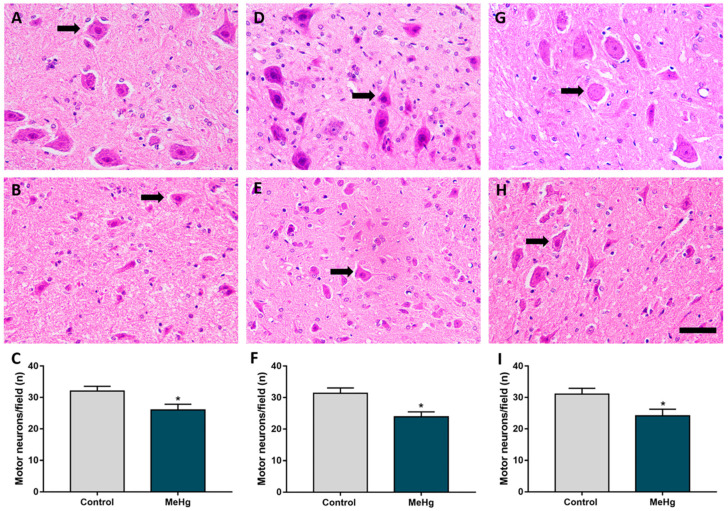
MeHg caused a reduction in the density of motoneurons in all areas evaluated. Effect of MeHg on the cervical (**A**–**C**), thoracic (**D**–**F**) and lumbar (**G**–**I**) segments of the spinal cord in the offspring rats (*n* = 7 animals per group) after exposure during the intrauterine and lactation periods to 40 μg/kg/day of MeHg (samples collected 21 days after 42 days of dosing). (**A**,**D**,**G**) are representative photomicrographs of the motoneuron counts in the control group and (**B**,**E**,**H**) in the exposed group. In (**C**,**F**,**I**) are the results of the motoneuron density, expressed as the mean ± standard error of the mean (*n* = 7–8 animals per group). * *p* < 0.05, Student’s *t*-test. Black arrows indicate motoneurons. Scale bar: 20 μm.

MeHg intoxication during the gestation and lactation periods induced loss of motor neuron cell bodies in the ventral horn of offspring rat SC (Figure 2). This neuronal loss occurred in all the analyzed SC segments (Figure 2B,E,H). There was a statistically significant reduction in motor neuron cell bodies in the SC of intoxicated animals compared with the control (Figure 2C,F,I). The average numbers per segment were as follows: cervical (control: 30.49 ± 1.57 *versus* MeHg: 26.02 ± 1.80; *p* = 0.01); thoracic (control: 31.38 ± 1.68; *versus* MeHg: 23.92 ± 1.52; *p* = 0.008); and lumbar (control: 31.06 ± 1.86 *versus* MeHg: 24.14 ± 2.11; *p* = 0.02) (Figure 2).

### 2.4. MeHg Exposure during Intrauterine and Lactational Periods Induces Downregulation of Myelin Basic Protein in Offspring Rats

**Figure 3:**
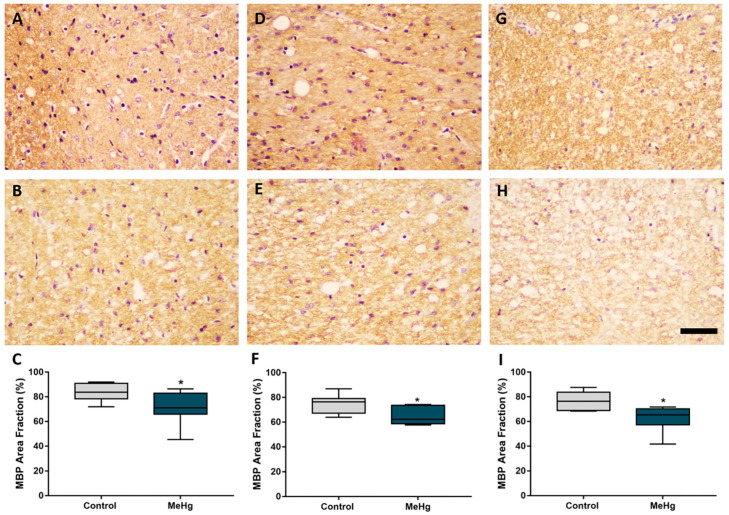
Exposure to MeHg caused a reduction in the immunomarked area in myelin basic protein in all areas evaluated. Effect of MeHg on the cervical (**A**–**C**)**,** thoracic (**D**–**F**) and lumbar (**G**–**I**) segments of the spinal cord in the offspring rats (*n* = 7 animals per group) after exposure during the intrauterine and lactation period to 40 μg/kg/day of MeHg (samples collected 21 days after 42 days of dosing). (**A**,**D**,**G**) are representative photomicrographs of the assessment of the fraction of area immunolabeled by anti-myelin basic protein (MBP) in the control group and (**B**,**E**,**H**) in the exposed group. Counterstaining with Harris hematoxylin. In (**C**,**F**,**I**) are the results of the MBP area fraction, expressed as the mean and interquartile deviation (*n* = 7–8 animals per group). * *p* < 0.05, Mann–Whitney test. Scale bar: 20 μm.

MeHg exposure during gestational and lactation periods induced conspicuous impairment in myelin basic protein immunoreactivity (Figure 3). Nonparametric analysis showed that there was a reduction in the fraction (%) of immunolabeled area in the cervical (control: med. 83.77 ± 12.63 inter. desv. *versus* MeHg: med. 71.15 ± 17.11 inter. desv.), thoracic (control: med. 76.44 ± 11.91 inter. desv. *versus* MeHg: med. 62.19 ± 14.86 inter. desv.) and lumbar (control: med. 76.33 ± 14.79 inter. desv. *versus* MeHg: med. 65.37 ± 13.02 inter. desv.) segments, which is perceptible by MBP staining (Figure 3).

## 3. Discussion

The results showed that continuous MeHg exposure of pregnant rats during pregnancy and lactation cause motor neuron loss and downregulation of myelin basic protein in the SC of offspring rats. These pathological events occur even after MeHg exposure cessation. It follows that the low dose exposure during the perinatal period is able of triggering a neurodegenerative pattern that persist after complete maturation of the CNS.

The CNS development starts during intrauterine life and reaches the peak of maturation in adulthood, being susceptible to drugs, substances of abuse and environmental toxicants [14,15,16]. Therefore, this period is crucial for the proper development of anatomical structures and molecular processes. Several studies tried to correlate neural development events between humans and rodents. Semple et al. [17] pointed out that rodents between 35 and 49 days of life correspond to events that occur in humans aged 12–18 years. Those events include the peak of synaptic density, white matter increase, and continuation of the myelination process. Considering the CNS susceptibility to xenobiotics, some observational studies had pointed out the association between prenatal MeHg exposure and neurological and hypertension outcomes [18,19], which reinforces the need for paying attention to those vulnerable populations, such as in Amazonian riverine areas.

As previously mentioned, the bioaccumulation of MeHg in fish consumed by humans poses a major threat to them. Crespo-Lopez et al. [2] have shown that people living in endemic regions of mercurial exposure may be submitted to mercury levels about three times higher than those recommended by the World Health Organization [1]. Thus, considering the reality faced by the population exposed to contaminated food with MeHg, our intoxication model, with 40 μg/kg/day, is representative because it corresponds to a prolonged period of systemic exposure.

This chosen dose has been extensively studied in the last years by our and other groups in different tissues, including somatosensory cortex, salivary glands, and alveolar bone [10,20,21,22,23,24,25,26]. Nevertheless, the effect of this dose regimen had not been investigated during pregnancy and lactation in the newborn rat SC. The data have shown that the total mercury levels in the blood of the exposed offspring had mean THg levels of 0.32 µg/mL, which clearly shows mercury bioavailability even after mercurial exposure cessation.

After crossing biological barriers, various studies point to the multiple mechanisms by which mercury can trigger cell damage, such as oxidative stress, genotoxicity, and damage to microtubules, which can lead to cell death [5,27]. We have found a conspicuous reduction in the cell density of motor neurons, indicating the susceptibility of newborn rats SC to continuous MeHg during the period of CNS formation and maturation. Such mercury -induced damage observed in the present study can be associated with an mercury mechanism related to oxidative stress and modulation of the proteomic profile in the SC [25].

The SC is composed of several cell types important for conducting nerve impulses through ascending and descending fibers that carry motor and sensory stimuli, such as interneurons and motoneurons [28], with motoneurons being the cells present in the gray matter responsible for the propagation of nerve impulses related to motility [9]. In this study, there was a reduction in the MBP immunoreactivity in all segments of the newborn rat SC. It has been previously shown that the SC is susceptible to the harmful effects of other pollutants, such as lead and inorganic mercury [29,30]. In adult rats, lead exposure is associated with a decrease in antioxidant capacity against peroxyl radicals, a reduction in the density of motoneurons in the three anatomical segments, and a decrease in MBP immunostaining in the thoracic and lumbar regions [30]. In addition, inorganic mercury also triggers biochemical and structural damage to the SC, through the modulation of proteins related to oxidative biochemistry and energy metabolism. In these studies, the same neurodegenerative pattern here described were also observed, including ultrastructural damage to the myelin sheath, which suggest that demyelination is a common event following MeHg intoxication [29].

Continuous MeHg exposure during gestation and lactation periods induces conspicuous damage to spinal motor neurons and myelin impairment in newborn rats. This suggests that SC is especially susceptible to mercury intoxication. The myelin demise might contribute to functional motor problems and impairs the conduction of nerve impulse. From a health outcome perspective, this may have significant impacts on the SC of people exposed to heavy metals and other environmental pollutants, especially children and adolescents who were exposed during the intrauterine period and CNS maturation phase.

## 4. Materials and Methods

### 4.1. Ethical Aspects

The present study was approved by the Ethics Committee on Animal Experimentation of the Federal University of Pará under protocol number 8613011217. The entire experiment followed the ARRIVE 2.0 guideline and the NIH Guide for the Care and Use of Laboratory Animals [31].

### 4.2. Experimental Animals

Female Wistar rats (*Rattus norvegicus*), aged 90 days and weighing 175 ± 25 g, were kept in individual cages in a controlled environment, with temperatures around 25 °C ± 2 °C, and with a light/dark cycle (lights on at 6 a.m. and off at 6 p.m.). The experimental protocol started one day (D1) after the pregnancy diagnosis made by the observation of a vaginal tampon (D0). The pregnant rats were kept in cages with food and water ad libitum. Malformation and malnutrition were considered exclusion criterion, although no exclusions were reported in the study.

### 4.3. MeHg Exposure Protocol

MeHg (Sigma Company, St Louis, MO, USA) was solubilized in 100% ethanol P.A. according to the dose calculation. The solution containing the toxicant was added to cookies (Teddy Grahams, Nabisco Ltd., East York, ON, Canada) that were left at room temperature for complete volatilization of ethanol and impregnation of MeHg in accordance with what was previously described by Kirkpatrick et. al. [32] and adapted by Nascimento et al. [27,33].

On the day after pregnancy diagnosis, rats were randomly divided into two groups: the MeHg (which received daily 40 μg/kg body weight, totaling 1.68 mg/kg over 42 days) and control (received the vehicle using the same treatment scheme). The dose was adapted from Kong et al. [34], in which they suggested that this concentration of MeHg is able to promote mercury systemic distribution and triggers biochemical and molecular alterations in brain regions. The animals were identified in sequential numbers by D.C.B.S and L.O.B., weekly weighed for dose adjustment with seven animals in each group according to protocol described by Nascimento et al. [33]. Using this protocol with long-term exposure at low doses, there is no maternal abandonment [27,33]. MeHg oral administration occurred, daily, during the gestation period (20–21 days) and during the lactation period (20–21 days), with the MeHg administration proceeded after dissolving MeHg chloride (CH_3_HgCl; Sigma Aldrich, Milwaukee, WI, USA) in ethanol, and then incorporating it into cookie treats (Teddy Grahams, Nabisco Ltd., East York, ON, Canada). In this protocol, the cookies were allowed to airdry overnight with controlled humidity to allow the ethanolic vehicle to evaporate, and the control cookies were treated with the ethanolic vehicle only. The cookies with MeHg were placed separately for each rat in the cage, making it possible to guarantee that each rat had consumed the pre-established dose added to the cookie. At the end of the MeHg administration, the newborn animals were kept in collective cages (4 animals each) with food and water ad libitum, in a controlled temperature and a 12 h light/dark cycle. Animals were euthanized at 42 days after treatment onset, a period associated with completed CNS maturation [17].

### 4.4. Sample Collection and Perfusion

**Figure 4:**
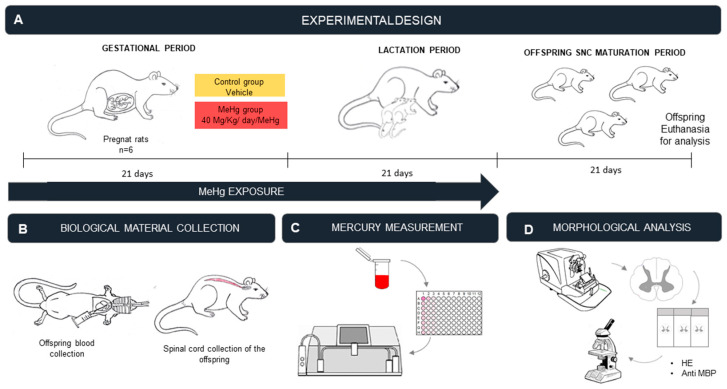
Methodological design: (**A**) description of the protocol for exposure of pregnant rats to MeHg; (**B**) collection of biological material from the offspring (blood and spinal cord); (**C**) quantification of mercury (Hg) in the blood; (**D**) analysis of neuronal density by counting of the motor neuron cell bodies in hematoxylin and eosin-stained sections, and evaluation of MBP immunoreactivity using immunohistochemistry.

At the end of the experimental period, intoxicated offspring rats were anesthetized with a mixture of ketamine hydrochloride (90 mg/kg) and xylazine hydrochloride (9 mg/kg). The blood was collected in a heparinized tube for analysis of THg. Animals were then perfused through the heart left ventricle with heparinized 0.9% saline solution followed by 4% paraformaldehyde in 0.1 phosphate buffer. After the spinal laminectomy, blocks of all the SC segments were collected (Figure 4).

### 4.5. Measurement of THg Levels in the Blood

A total of 500 μL of a blood sample was digested on a hot electric plate (200–300 °C) for 30 min using solution of nitric acid (HNO_3_), perchloric acid (HClO_4_) and sulfuric acid (H_2_SO_4_) at a concentration of 1:1:5 *v*/*v*. The measurement of mercury concentration was performed with cold vapor atomic absorption spectrophotometry (Mercury semi-automatic analyzer-Hg 201, Sanso Seisakusho Co. Ltd., Tokyo, Japan). Mercury was first converted into elemental mercury vapor in order to be introduced into an absorption and quantification cell. The mercury concentration was expressed in μg/mL (see more details in Chemelo et al. [35]).

### 4.6. Histological Procedures

The spinal cord samples were post-fixed in Bouin’s solution for 12 h, dehydrated in increasing alcohol solution, clarified in xylene and embedded into paraffin. Transversal sections of 7 μm were obtained from the cervical, thoracic and lumbar segments using a microtome. Sections were mounted onto microscopy slides for histochemistry and immunohistochemical analyses.

#### 4.6.1. Counting of Motor Neurons

For staining of motor neurons, 7 μm sections were mounted onto microscopy slides and kept in an oven for at least 24 h at 60 °C for deparaffinization. The routine HE staining technique was performed. Slides were surveyed on a bright field optical microscope (DS-Fi3 microscope with camera attached to the Nikon Eclipse Ci H550s bright-field microscope). More representative images were digitalized for the counting procedures.

Motor neurons were clearly observed in the ventral horn of rat SC. Motor neuron identification was performed according to morphological parameters described by Ferucci et al. [36], which included large, multipolar basophilic cells that have nuclei with little condensed chromatin. Only motor neurons located at SC ventral horns were included in the analysis. Cell counting was performed using NIH ImageJ software, version 1.52. Both SC sides were analyzed in the cervical, thoracic and lumbar regions in the control and intoxicated animals.

#### 4.6.2. Assessment of Immunoreactivity of Myelin Basic Protein

For immunolabeling of the myelin sheath, we used an antibody against MBP [37,38]. Mounted sections were previously prepared with poly-d-lysine for better adhesion. The slides were kept in an oven for at least 24 h at 60 °C. Immunohistochemistry was performed according to the experimental protocol adapted from Bittencourt et al. [22]. Slides were deparaffinized, hydrated and immersed in phosphate buffer saline (PBS) before incubation in a citrate buffer at 70 °C for 25 min for antigenic retrieval. Sections were then immersed in PBS for 10 min, followed by treatment in a methanol–hydrogen peroxide solution (3:100, *v*/*v*). Anti-MBP antibody (1:100, Chemicon International, Temecula, CA, USA) was used, the second most abundant protein in the composition of the myelin sheath and fundamental for the organization of the lamellae [39]. Sections were 3,3′diaminobenzidine (DAB) reacted, and counterstained with Harris hematoxylin, then dehydrated in alcohol, cleared in xylene, and coverslipped with Entellan (Merck, Darmstadt, Germany) [40]. Sections were then observed using a 40× objective on a bright-field optical microscope (DS-Fi3 microscope camera attached to the Nikon Eclipse Ci H550s bright-field microscope). Digitalized images of more representative fields were obtained. The determination of the fraction of area immunolabeled with anti-MBP was carried out according to a previous protocol published by our group [30,41] using ImageJ software with the Color Deconvolution plugin.

### 4.7. Statistical Analysis

The results were analyzed by GraphPad Prism 7.0 software (GraphPad Software Inc., La Jolla, CA, USA). To verify normality, the Shapiro–Wilk method was used. Statistical analysis was performed using a Student’s *t*-test for parametric data (tHg levels and MN count) and Mann–Whitney test for non-parametric data (immunolabeled area fraction). The test’s power was calculated in OpenEpi (OpenEpi 2.3.1, Emory University, Atlanta, GA, USA) using the difference between the two means, power of 80% and with a type I error of 5%. The results are expressed as the mean ± standard error of the mean (SEM) when parametric and the median (med.), represented as the medians (medi.) and interquartile deviation (inter. desv.), when not parametric. A *p* < 0.05 was adopted as statistically significant.

## 5. Conclusions

In conclusion, our findings provide strong evidence that exposure of pregnant rats during the gestational and lactation periods to MeHg increases the bioavailability of tHg in the blood of the offspring, contributing to motor neuron loss in the ventral horn of SC, as well as myelin impairment in the white matter tracts. Further studies are needed to elucidate the specific mechanisms by which these neuropathological findings occur as well as the susceptibility of other neuronal and glial cell populations.

## Data Availability

All data are available within the article.

## Abbreviations

MeHg	methylmercury
MBP	myelin basic protein
Hg^0^	elemental Mercury
HgCl_2_	mercury chloride
CNS	central nervous system
SC	spinal cord
THg	total mercury
PB	phosphate buffer
HNO_3_	nitric acid
HClO_4_	perchloric acid
H_2_SO_4_	sulfuric acid
PBS	phosphate buffer saline
DAB	3,3′diaminobenzidine
